# Bacterial Translocation – Impact on the Adipocyte Compartment

**DOI:** 10.3389/fimmu.2013.00510

**Published:** 2014-01-06

**Authors:** Tassilo Kruis, Arvind Batra, Britta Siegmund

**Affiliations:** ^1^Department of Medicine I (Gastroenterology, Rheumatology, Infectious Diseases), Charité – Universitätsmedizin Berlin, Berlin, Germany

**Keywords:** Crohn’s disease, mesenteric fat, innate receptors, adipokines, bacterial translocation

## Abstract

Over the last decade it became broadly recognized that adipokines and thus the fat tissue compartment exert a regulatory function on the immune system. Our own group described the pro-inflammatory function of the adipokine leptin within intestinal inflammation in a variety of animal models. Following-up on this initial work, the aim was to reveal stimuli and mechanisms involved in the activation of the fat tissue compartment and the subsequent release of adipokines and other mediators paralleled by the infiltration of immune cells. This review will summarize the current literature on the possible role of the mesenteric fat tissue in intestinal inflammation with a focus on Crohn’s disease (CD). CD is of particular interest in this context since the transmural intestinal inflammation has been associated with a characteristic hypertrophy of the mesenteric fat, a phenomenon called “creeping fat.” The review will address three consecutive questions: (i) What is inducing adipocyte activation, (ii) which factors are released after activation and what are the consequences for the local fat tissue compartment and infiltrating cells; (iii) do the answers generated before allow for an explanation of the role of the mesenteric fat tissue within intestinal inflammation? With this review we will provide a working model indicating a close interaction in between bacterial translocation, activation of the adipocytes, and subsequent direction of the infiltrating immune cells. In summary, the models system mesenteric fat indicates a unique way how adipocytes can directly interact with the immune system.

## Working Model

### What is inducing adipocyte activation?

Classically, adipose tissue was categorized as the site of energy storage and release. But this concept changed profoundly within the last decades when adipose tissue has been identified as a potent endocrine organ with effects on metabolism and immunity. Fat tissue is a powerful producer of fatty acids, classical cytokines, chemokines, and adipokines, to name just some of the factors released. Considering that adipose tissue accounts for 10–20% of the body weight in males and 20–30% in females (1) one can well accept that this endocrine tissue has been studied more closely for its impact on body homeostasis and immune cell regulation.

Adipose tissue comprises not only mature adipocytes but harbors a variety of other cells including adipocyte precursor cells (preadipocytes), fibroblasts, and immune cells including T cells and macrophages. The size of adipocytes varies according to their lipid content and is linked to the function of the cells, with larger adipocytes usually being more active with regard to their metabolic activity and adipokine production (2). In mammals two kinds of adipose tissue exist. Brown adipose tissue plays an important role in thermogenesis in particular in neonates whereas the main adipose tissue present in adults belongs to the white adipose tissue. Hence this review will focus on white adipose tissue, which is distributed throughout the body mainly at two different sites, the subcutaneous and the visceral compartment. Subcutaneous fat is present as hypodermis under the skin and visceral adipose tissue surrounds organs in the abdominal cavity. Both compartments exert different competence with regard to metabolic and immune regulating function with the increase in metabolic diseases in particular being linked to the visceral fat (3). The visceral fat can be subdivided into omental fat which surrounds the intestine superficially, retroperitoneal fat near the kidney and the mesenteric fat in close proximity to the intestine (4).

In Crohn’s disease (CD), a specific hypertrophy of the mesenteric fat adjacent to the inflamed intestinal segments occurs. Burrill J. Crohn himself reported in the thirties of the last century, that mesenteric fat is increased and closely attached to inflamed parts of the colon and termed this observation “creeping fat,” an alteration unique to CD patients. Since then this initial report was verified by different techniques and groups over the last years (5–7). CD is a chronic intestinal inflammation that mainly affects the terminal ileum and the proximal large bowel, but can potentially involve any location of the alimentary tract. The etiology of the disease is not finally understood, and current therapeutic strategies are not curative, although they are effective in alleviating clinical symptoms.

But even though the close contact of creeping fat and inflamed intestinal segments in CD has been known for decades, the role of this fat hypertrophy and the consequences of this local adipose tissue increase for the ongoing intestinal inflammation are not understood yet (8). With the perception regarding the multiple functions of adipose tissue changing in the recent years, the interest in deciphering the link between the mesenteric fat hypertrophy in patients and the intestinal inflammation arose. Even though the interaction between the sites is still not completely understood, there is solid evidence, that they affect each other.

It is not known during which stage of disease the mesenteric fat hypertrophy develops, but one underlying cause expected to be related to the local adipose tissue remodeling is the altered intestinal barrier function and subsequent bacterial translocation. In CD, as well as in experimental models of intestinal inflammation, such increased translocation of bacteria into the intestinal fat occurs. This finding is already leading to our first questions: how does inflammation arise in mesenteric adipose tissue in the setting of CD?

Bacterial translocation represents a possible mechanism as altered epithelial permeability is a feature of CD disease and bacteria are more frequently present in both mesenteric adipose tissue and mesenteric lymph nodes of CD patients (9, 10). Similarly, in experimental models of inflammatory bowel disease (IBD) such as dextran sulfate sodium (DSS)-induced colitis or indometacin-induced ileitis bacterial translocation was enhanced (10, 11).

But one has to bear in mind that bacterial translocation is not unique to states of intestinal inflammation, however it is increased when compared to healthy subjects where bacterial translocation to mesenteric adipose tissue can equally be detected (10).

Concerning the mechanisms leading to increased permeability in intestinal inflammation several factors are discussed for bacteria. First, the intestinal barrier consists of the secreted mucus barrier as well as the cellular barrier (12). The barrier formed by mucus and here in particular the dysregulation of defensin production plays a critical role in CD (13–15). Thus targeting alterations in the composition of these anti-microbial peptides might serve as therapeutic strategy in the future (16).

In addition, defects in the cellular barrier have been reported. Söderholm and colleagues detected augmented tight junction permeability in the intestine of CD patients (17). Zeissig and colleagues linked changes in the expression of claudins 2, 5, and 8 to reduced strands of tight junctions in active disease accompanied by an increased rate of epithelial cell apoptosis in active CD (18). While the increased incidence of bacterial translocation in CD patients is commonly accepted, the mechanisms allowing for bacterial translocation are not finally dissected. In fact, the underlying mechanisms might even be different in ileal and colonic CD, as depicted in detail by Gersemann and colleagues in a recent review (16). Leading us to the question what might be the consequence of increased bacterial translocation into the adjacent adipose tissue?

Besides their energy storing capacity and their secretory activity, adipocytes share commonalities with immune cells. An initial link between adipocytes and innate immunity was a report, that depicted the plasticity of murine preadipocytes which could convert to macrophage-like cells, characterized by the expression of macrophage markers like F4/80 and Mac-1 as well as co-stimulatory molecules including CD80 and CD86 (19). In the meantime further similarities have been revealed. For instance, macrophages, preadipocytes, and adipocytes express different classes of pattern recognition receptors enabling them to respond to microbial moieties with either increased or decreased secretion of immunological active molecules (11, 20, 21).

#### Adipose tissue responsiveness to endotoxin

Already in the seventies of the last century it was shown that adipose tissue is responsive to bacterial compounds such as endotoxin as assessed by alterations in lipid metabolism in rodents following injection of this bacterial antigen with differences in the grade of lipolysis between subcutaneous and mesenteric fat (22–24). This sensitivity of fat toward bacterial components was well studied during the following decades, but again with an emphasis on the effects on metabolism (25, 26).

Taking into account that the availability of energy is a prerequisite for immune function, in the following years the consequences of adipose tissue stimulation by bacterial products for the immune system were studied. Local LPS application induced the release of glycerol from adipose tissue surrounding lymph nodes, thus supporting the initiation of the immune response and linking the capacity of adipose tissue to detect bacterial products (27, 28). Again, these studies mainly focused on the biological effects of stimulation and not on the mode of antigen recognition involved.

#### Toll-like receptors

In 2000 Lin and coworkers demonstrated that adipocytes express Toll-like receptors (TLR) (29), a group of transmembrane receptors that sense highly conserved microbe-associated molecular patterns (30).

So far a total of 13 TLR have been identified, with 10 and 12 functional in humans and mice (31). These receptors recognize conserved structures that are not present in the host, but more or less frequently present in viruses, fungi, or bacteria and include for example zymosan, flagellin, nucleic acids, and LPS (32).

Stimulation of these innate receptors by their specific ligands is followed by the activation of signaling pathways such as nuclear-factor kappaB (NFκB) or mitogen-activated protein kinase (MAPK), resulting in the production of pro-inflammatory cytokines in immune cells (33). Lin and colleagues described not only the expression of TLR on adipocytes and preadipocytes but further revealed that the activation of TLR4 resulted in an increase of TLR2 expression in adipocytes, already suggesting that the response to bacterial products is well orchestrated in these cells (29).

Further studies confirmed that adipocytes and preadipocytes express numerous receptors from the TLR family. We and others have provided evidence for the functional expression of TLRs on adipocytes and preadipocytes. For example stimulation of TLR4 in adipocytes and preadipocytes by LPS induced increased production of classical cytokines and chemokines including IL-6, MCP-1, and TNFα (34–36).

When characterizing TLR expression and responsiveness, our group revealed that leptin deficiency, an adipokine initially described as a satiety signal, altered TLR expression and cytokine release following stimulation (35). This observation was confirmed by Kim and colleagues who reported an increased TLR expression not only in leptin-deficient mice, but equally in mice fed a high-fat diet (37). This link between leptin and TLR expression could be confirmed in human cells where expression of TLR2 on monocytes is enhanced in the presence of high levels of leptin. An effect, that might contribute to the increased immune response seen in obesity (38). Furthermore, this observation is bridging the energy status of the organism to the initial concept, that responsiveness of adipose tissue to bacterial antigens is linked to the regulation of energy supply.

In fact the interplay of energy supply or nutrition and TLR expression in adipocytes forms a subject of recent research in the field of metabolic diseases. For example supply of citrus flavonoid is not only beneficial in obesity-related diseases but was furthermore linked to its’ modulating effects on TLR2 expression (39). Bearing such mutual interactions between energy supply and TLR expression in mind it seemed a logical consequence to ask whether pathogen-associated molecular patterns present the sole ligands for TLR?

One group reported in 2009, that at least TLR4 on adipocytes can be stimulated directly by fatty acids leading to the activation of NF-κB with different fatty acids inducing specific profiles of released adipokines (40). Indeed it was an intriguing thought, that in altered states of fatty acid release by the adipose depot, i.e. in various states of nutritional supply, the TLR signaling pathway is involved in the modulation of the endocrine activity of the fat tissue compartment. However, in 2009 this view was negated by Erridge and Samani who pointed out that these studies dealing with TLR4-specific stimulation following supply of fatty acids *in vitro* might be hampered by LPS contamination of the fatty acids employed or the proteins used for solving them, thus questioning direct effects of the fatty acids on TLR stimulation (41). This later view is supported by a study that investigated fatty acid effects on TLR activation where no direct stimulation of TLR2 and TLR4 by dietary saturated and unsaturated fatty acids was measured (42).

Not only the direct effect of altered fatty acid levels in adipose tissue in the course of diet or infection on TLR has not been deciphered yet. Conflicting reports about the link between the nutritional status and the TLR expression exist. While recently one report stated that mRNA levels of TLR2, TLR6, and TLR7 are decreased in mice fed a high-fat diet (43), other studies provide evidence that TLR1-9 and TLR11-13 are up-regulated in murine adipose tissue following obesity-induction by a high-fat diet (37). Thus deciphering the impact of TLR expression on the function of adipose tissue cells is still a field full of open questions that requires further clarification. However, there is an additional class of receptors present in adipocytes that further supports their commitment to the innate immune system, the so-called nucleotide-binding oligomerization domain (NOD)-like receptors.

#### The nucleotide-binding oligomerization domain-like receptors

Like TLR, NOD-like receptors detect highly conserved non-self antigens. This group comprises the subfamilies of NOD, NLR family pyrin-domain-containing proteins (NLRP), NLR family CARD-domain containing (NLRC), neuronal apoptosis inhibitor factors (NAIP), NLRX, and MHC II transactivator (CIITA), with all of these receptors being localized in the cytosol (32, 44). NOD1 and NOD2 were the first members of the NLR family to be described and are responsive to subunits of peptidoglycans (45, 46). In CD these receptors gained particular interest, since polymorphisms in *NOD2* were linked to an increased risk of developing CD (47, 48). Our group was the first to show that preadipocytes express not only NOD1 and NOD2 receptor specific mRNA but functional cytosolic receptors (20). Since then, several groups confirmed the presence of functional NOD receptors in fat cells from mice and man and postulated both, effects on immune response and insulin sensitivity as a consequence of stimulation (49, 50).

A recent study depicts that NOD activation is linked to adipocyte differentiation. In a murine cell line differentiation of preadipocytes to mature adipocytes was decreased by NOD1- but not NOD2-specific stimulation. The authors noticed some species specific differences when they tested this observation in human cells where activation of either of the two receptors, NOD1 or NOD2, suppressed maturation of adipose tissue derived stem cells to adipocytes (51).

Even though it is well accepted that genetic variants of NOD2 are associated with a higher susceptibility to CD (52) the consequence of NOD2 stimulation by bacterial products is not finally understood. Our own group noticed regulation of NOD2 mRNA in preadipocytes following stimulation of NOD1 or via pro-inflammatory cytokines. But unlike stimulation of NOD1 no induction of cytokine release took place thereafter (20). This is in line with the current concept, that NOD2 activation might be a regulator of TLR-induced cytokine production. For example in human dendritic cells loss of functional NOD2 results in an increased IL-23 production induced by stimulation with *E. coli* (53). In a study published this year, the effect of the *NOD2* variants on bacterial translocation was characterized in patients. As compared to controls bacterial mRNA was more frequent in the blood of patients carrying either a NOD2- or an ATG16L1-variant genotype. In addition, the presence of bacterial DNA was related to disease activity and in patients with *NOD2* variants the phagocytic and bactericidal activity of blood neutrophils was decreased (54). Even though this study omitted any information about mesenteric adipose tissue it is tempting to speculate that variants of CD susceptibility genes might alter responses therein to bacterial products. But what mechanisms can be activated in adipocytes following the stimulation of the innate receptors? As mentioned before, adipose tissue is the source of a plethora of mediators which will be presented together with information about the biological consequences of the release in the next section.

### Which factors are released by adipocytes after activation and what are the consequences for the local fat tissue and macrophages compartment

The infiltration of mesenteric adipose tissue by macrophages was initially described in murine models of obesity (55, 56), an observation subsequently confirmed in humans (57, 58). About 30% of transcripts in mesenteric adipose tissue that correlated strongest with obesity were inflammatory or macrophage specific (55). The infiltration of macrophages into the inflamed mesenteric adipose tissue represents a cardinal feature of creeping fat in CD (59). Although fat wrapping is not typically observed in animal models of colitis, macrophage infiltration of mesenteric adipose tissue contiguous to sites of intestinal inflammation has been described (60, 61).

Activation of the mesenteric adipose tissue results in the release of cytokines (e.g., IL-6, TNFα), chemokines (e.g., MCP-1/CCL2, Rantes/CCL5), adipokines (e.g., leptin, adiponectin, resistin, visfatin), but also fatty acids (26, 62–64). Furthermore, preadipocytes have the potential to differentiate into macrophage-like cells that express macrophage markers like F4/80, Mac-1, CD80, or CD86, and are capable to phagocytose microorganisms (19). In mesenteric adipose tissue of patients with CD a variety of such potent molecules can be detected (59, 65–67) thereby influencing macrophage recruitment and polarization.

In parallel to T helper cells the concept of macrophage polarization to either classically activated M1 macrophages or alternatively activated M2 macrophages was established. M1 macrophages produce pro-inflammatory cytokines such as IL-1β, IL-6, and TNFα, as well as effector molecules including reactive oxygen species or nitric oxide. In contrast, M2 macrophages express high amounts of IL-10, scavenger-, mannose-, and galactose-type receptors, are involved in Th2 cell activation and regulate extracellular matrix molecule synthesis, wound repair, and tumor progression. However, it is well accepted that *in vitro* generated M1 or M2 macrophages represent extreme states on a continuum with tissue macrophages displaying phenotypes comprising hallmarks of both (68–70). Adipose tissue macrophages are characterized by the expression of M2 surface markers like CD163, CD206, CD209, and CD200, a high phagocytic activity, and the secretion of IL-10 and IL-1 receptor antagonist (IL-1Ra). They release pro-inflammatory molecules including TNFα, IL-6, IL-1, MCP-1, and MIP-1α in quantities higher than *in vitro* generated M1 macrophages. These macrophages cluster around necrotic adipocytes indicating a functional role in the clearance of extracellular lipid and cellular debris while they also contribute to adipose tissue inflammation and metabolic dysfunction by inflammatory cytokines (71, 72). In obesity the adipose tissue macrophage phenotype is skewed even further toward M1 polarization (73, 74), whereas in CD adipose tissue macrophages show an increased expression of M2 markers (75).

In the following, we will discuss molecules that are secreted by activated preadipocytes or adipocytes under inflammatory conditions with the potential to recruit macrophages and modulate their phenotype or function. If LPS was used as an activating stimulus, it was derived from *E. coli*.

#### IL-6 and TNFα

Both IL-6 and TNFα are key players in inflammation and their role in the pathophysiology of CD is well established. IL-6 serum levels correlate with disease severity and localization (76). Estimated 10–30% of circulating IL-6 is released from adipose tissue (77). Increased TNFα levels have been observed in serum and intestine of CD patients, more important, neutralizing TNFα represents an approved treatment (78–81). Both IL-6 and TNFα are released by preadipocytes and adipocytes upon stimulation with LPS (62, 63). Regarding IL-6, its expression is increased in the mesenteric adipose tissue in TNBS colitis and inflammation is accompanied by macrophage infiltration (60, 61). However, in inflamed mesenteric adipose tissue of CD patients IL-6 expression seems not to be significantly higher than in other inflammatory conditions like colorectal cancer (CC) or diverticulitis (67, 82).

IL-6 exerts direct chemotactic effects on monocytes by inducing integrin expression, cell attachment, and transmigration through the endothelium (83–85). Whether IL-6 itself modulates macrophage phenotype and function is not reported yet. But in preliminary studies we observed that at least *in vitro* the presence of IL-6 directs macrophages to an anti-inflammatory phenotype characterized by expression of CD163 and release of IL-10 (unpublished data). However, IL-6 might influence the polarization of adipose tissue macrophages via regulation of adipokine secretion. For example, IL-6 in combination with soluble IL-6 receptor inhibits adiponectin expression in 3T3-L1 adipocytes and primary human adipocytes. Similarly, TNFα down-regulates adiponectin secretion paralleled by an increase in leptin expression (86–88). The impact of TNFα signaling on macrophage polarization is supported by studies in TNF-receptor 1 (TNFR1) and TNFR2 deficient mice. On a high-fat diet, they show increased weight gain and adipose tissue macrophage infiltration. Surprisingly, the expression of pro-inflammatory cytokines like IL-6 and MCP-1 is reduced paralleled by a skew of adipose tissue macrophages from M1 to M2 polarization (89).

A further indirect evidence that TNFα is of significance for macrophage polarization is the observation that the anti-TNFα antibodies infliximab and adalimumab induce M2-like macrophages in a mixed lymphocyte reaction. These cells inhibit T-cell proliferation, secrete IL-10, and display an up-regulation of the M2 marker CD206. However, the observed effect is probably not only mediated by neutralizing TNFα but also by Fcγ-receptor mediated binding of TNFα-antibody complexes (90).

#### C-reactive protein

The acute phase reactant C-reactive protein (CRP) is widely used in clinical routine as a biomarker of inflammation in both infectious and autoimmune diseases. In CD serum CRP levels do not only indicate acute inflammation but also have predictive value regarding the course of disease (91). While liver is considered to be the main source of CRP (92) evidence is mounting that adipose tissue is another important source. Circulating CRP levels increase with BMI in obese patients (93) while in CD CRP levels correlate with mesenteric fat density measured by CT enterography (5). Recently, elevated CRP expression was demonstrated within mesenteric adipose tissue of CD patients compared to healthy controls and patients with ulcerative colitis (UC). Adipose tissue expression and plasma levels correlated indicating that adipose tissue might be an important source of total circulating CRP. Interestingly, this elevated CRP expression was paralleled by increased bacterial translocation to mesenteric adipose tissue and lymph nodes. *In vitro*, adipocytes release CRP after stimulation with TNFα, IL-6, LPS, and *E. coli*, but not Pam3Cys (a TLR2 ligand), MDP (a NOD2 ligand) or *Lactobacillus* spp. Thus, local cytokines and translocalized gram-negative bacteria may synergize to promote CRP expression in mesenteric adipose tissue (10, 93).

C-reactive protein binds to cell wall components of different bacteria, damaged cells, and nuclear autoantigens. In consequence, the classical complement cascade is activated leading to opsonization of the bound antigens. Furthermore, CRP itself binds to Fcγ-receptors on immune cells like monocytes and macrophages (92). Regarding its effect on monocytes and macrophages conflicting results were published. It was reported that CRP in synergy with LPS induces both pro-inflammatory and anti-inflammatory cytokines like IL-1β, IL-6, TNFα (94), and IL-1Ra in human monocytes (95, 96). For human alveolar macrophages both stimulatory and inhibitory effects of CRP on the release of IL-1α, IL-1β, TNFα, and IL-1Ra were described (95, 97). Incubation of human monocyte-derived macrophages or isolated rat macrophages with human CRP followed by LPS stimulation resulted in a decreased IL-10 secretion (98), whereas in mice treatment of macrophages with human CRP induced IL-10 production (99). This might be due to species differences in the role of CRP as an acute phase reactant.

Regarding macrophage polarization, incubation of human monocytes with CRP for 7 days resulted in pro-inflammatory macrophages characterized by a high expression of TNFα, IL-12, CCR2, MCP-1, and IL-1. In parallel less cells expressed M2 markers like CD206, CD163, and IL-10 even if primed before with IL-4 (100). On the contrary, it was described that CRP treatment of human monocyte-derived macrophages induced M-CSF release, a factor well known to polarize macrophages to an M2 phenotype (101). These discrepancies will have to be addressed in the future.

#### MCP-1 and RANTES

RANTES/CCL5 and MCP-1/CCL2 are both chemokines secreted from preadipocytes or adipocytes upon stimulation with LPS (62–64). Whereas RANTES/CCL5 is up-regulated in mesenteric adipose tissue of CD, MCP-1 is elevated in tissue from obese patients (66, 67, 102).

MCP-1 is an important macrophage chemoattractant in several inflammatory conditions and various tissues (103, 104). Evidence in the literature suggests that MCP-1 and its receptor CCR2 are relevant factors for macrophage recruitment and accumulation in inflamed adipose tissue. For instance, genetic ablation of MCP-1 or its receptor resulted in a decrease in obesity-induced adipose tissue inflammation and macrophage infiltration, the opposite was observed for MCP-1 over-expression (105–107). However, data on this topic are conflicting as other reports did not describe such an effect in MCP-1 knock-out mice arguing that MCP-1 is at least not critical for macrophage infiltration to adipose tissue (108, 109).

In murine tumor models blockage of MCP-1 does not affect overall tumor associated macrophage number, but results in a skew of macrophage phenotype with reduced expression of M2 markers (CD206 and arginase 1) and slight up-regulation of the M1 marker inducible nitric oxide synthase (iNOS) (110). Together with IL-6, MCP-1 favors survival of human CD11b^+^ peripheral blood mononuclear cells (PBMC) and augments CD206 expression as well as the total number of CD14^+^/CD206^+^ cells an effect mediated via inhibition of caspase-8 cleavage and enhanced autophagy (111). Besides MCP-1, RANTES and its receptor CCR5 may be involved in adipose tissue macrophage recruitment and polarization. For example, CCR5 knock-out mice display reduced numbers of adipose tissue macrophages accompanied by a skew toward an M2 phenotype (112).

#### M-CSF and GM-CSF

M-CSF is expressed significantly higher in mesenteric adipose tissue from patients with CD compared to diverticular disease and CC and its over-expression is associated with adipocyte hypertrophy (67, 113). Studies applying M-CSF knock-out mice indicate its importance for normal macrophage differentiation in various tissues (114). Regarding macrophage polarization, highly pure subsets of anti-inflammatory M2 macrophages can be obtained by differentiating human mononuclear cells in the presence of M-CSF (115). These cells stably express CD163 and IL-10 as a signature cytokine even in presence of strong co-stimuli such as IFNγ or CD40L. They release only relatively low amounts of pro-inflammatory cytokines like TNFα, IL-1β, IL-6, and fail to secrete IL-12/IL-23 (115, 116). This is accompanied by a slower IκBα degradation and recruitment of NFκB to its nuclear binding sites (117).

At least *in vitro*, opposite effects can be observed for GM-CSF polarizing human macrophages toward an M1 phenotype (115–118). Both M-CSF and GM-CSF seem to act as opponents on human macrophage function as they suppress the cellular response of each other when added to the same monocyte/macrophage population (119, 120). Whether GM-CSF expression is altered in mesenteric adipose tissue in CD has not been investigated yet. In murine obesity models, GM-CSF seems to be relevant for macrophage recruitment to mesenteric adipose tissue where it is highly expressed. GM-CSF knock-out mice display reduced numbers of adipose tissue macrophages accompanied by lower levels of pro-inflammatory cytokines (121). Thus, the local M-CSF/GM-CSF ratio might be a factor modulating macrophage polarization during inflammation. A high local expression of M-CSF might add to the enrichment of CD163^+^ M2 macrophages observed in the creeping fat of CD (75).

#### Leptin, adiponectin, resistin, and visfatin

The adipokine leptin is up-regulated in the mesenteric adipose tissue of CD and UC patients compared to CC and diverticular disease (65, 67). In contrast to other adipokines like adiponectin, resistin, and visfatin, which are all down-regulated leptin expression is not affected or even enhanced in long term LPS-activated adipocytes (64). Leptin seems to have direct effects on intestinal barrier function. Increased levels affect mucin production and increase colonic tight junction permeability (122–124). Furthermore it promotes growth of colonic epithelial cells (125). All these factors contribute to the integrity of the intestinal barrier hence leptin deficiency results in an increased bacterial translocation to the blood (126). Regarding its effects on monocytes and macrophages, leptin promotes phagocytosis and release of pro-inflammatory cytokines (e.g., IL-6, TNFα) and reactive oxygen species (127–129). Furthermore, it activates vascular endothelial cells resulting in up-regulation of adhesion molecules thereby facilitating diapedesis of monocytes (130). Data from our group and others indicate that leptin also modulates macrophage polarization. Culturing human monocytes in the presence of leptin results in an increase in the expression of the M2 surface markers CD14, CD206, and CD209 accompanied by relatively high expression of TNFα, IL-6, IL-1β, and IL-10 (131). Similarly, we observed an increased secretion of TNFα, IL-6, and IL-10 after leptin stimulation of *in vitro* generated M1 and M2 macrophages using GM-CSF or M-CSF, respectively. This was paralleled by an enhanced phagocytic and chemotactic capacity (75). Besides, leptin induces GM-CSF secretion at least in murine peritoneal macrophages that might affect their polarization in an autocrine manner (128). Taken together, leptin effects could contribute to the particular phenotype of adipose tissue macrophages displaying hallmarks of both M1 and M2 polarization (72).

Adiponectin belongs to C1q/TNF molecular superfamily and shares structural similarities with TNFα, TNFβ, and CD40L (132, 133). Yamamoto et al. reported that adiponectin expression was significantly increased in the creeping fat of patients with CD nearby inflamed intestine (134). Similarly, Paul et al. detected increased adiponectin expression in CD creeping fat (67). *In vitro* adiponectin expression is not altered in mature adipocytes stimulated by LPS. However, differentiation of preadipocytes to adipocytes is inhibited with a consecutive reduction in adiponectin expression (64). The effect of adiponectin on macrophages seems to be anti-inflammatory as it suppresses LPS induced TNFα and IL-6 release, increases IL-10 secretion and impairs the phagocytic capacity (135, 136). Adiponectin inhibits several pro-inflammatory pathways elicited by LPS, TNFα, and IL-6 in human macrophages via the induction of anti-inflammatory proteins (137). In creeping fat adiponectin levels correlated negatively with IL-6 and CRP expression (134). Opposite to leptin (130), adiponectin inhibits TNFα induced up-regulation of adhesion molecules like VCAM-1, ICAM-1, and E-selectin on vascular endothelial cells thereby preventing monocytes from attachment (138, 139).

Several studies investigated the modulating effects of adiponectin on macrophage polarization but with contradictory results. Adiponectin has been identified to induce M2 markers such as CD163, CD206, and CCL18 in both murine and human macrophages (140, 141). In RAW264.7 macrophages, adiponectin led to an M2 phenotype via an IL-4 dependent manner (142). In contrast to these data, the analysis of an adiponectin-induced transcriptome in human mononuclear cell-derived macrophages showed an augmented expression of 28 of 46 strictly M1-associated markers, whereas only 3 of 43 M2-associated markers were increased (143). In the latter study both M1 cytokines like TNFα and IL-6 and M2 cytokines such as CCL18 and CCL23 were induced. Data from our group indicate that adiponectin alters the phenotype and function of M1 and M2 macrophages differentiated with either GM-CSF or M-CSF, respectively, with the M2 phenotype being more susceptible. Adiponectin up-regulates IL-10, IL-6, TNFα, and CD206 expression in M2 macrophages thus leading to cells comprising both pro- and anti-inflammatory potency as described for adipose tissue macrophages (75).

Circulating resistin levels are increased in IBD (144, 145). Increased resistin expression was found in the mesenteric adipose tissue of patients operated on CD and diverticulitis but not CC (67). In human adipose tissue, resistin expression is 2.5 times higher in the omental than in subcutaneous fat with non-fat cells being its main source (146, 147). In human PBMC and macrophages, resistin expression can be induced by different stimuli such as TNF-α, IL-1, IL-6, and LPS (148, 149). But also LPS stimulation of isolated human subcutaneous adipocytes results in increased resistin expression (150). Furthermore, experimental endotoxemia causes a strong rise in circulating resistin levels (149). Resistin itself induces the secretion of pro-inflammatory cytokines such as IL-1β, IL-6, IL-12, and TNFα in human PBMC and macrophages (151, 152). Furthermore, it up-regulates adhesion molecules like ICAM-1 and VCAM-1 as well as MCP-1 in human endothelial cells facilitating monocyte recruitment (153). However, whether resistin directly modulates macrophage polarization has not been investigated to our knowledge.

Visfatin is an adipokine preferentially expressed in visceral adipose tissue (154). Its circulating levels are increased in IBD positively correlating with disease activity (145, 155, 156) but also in other inflammatory conditions such as acute lung injury (157), atherosclerosis (158), and rheumatoid arthritis (159). In colonic biopsy specimens of IBD patients, adipocytes as well as CD163^+^ macrophages from both submucosa and mesenteric adipose tissue were identified as cellular sources of visfatin. In human CD14^+^ monocytes, visfatin induced both pro- and anti-inflammatory cytokines including IL-1β, IL-6, TNFα, IL-1Ra, and IL-10, respectively. In GM-CSF differentiated macrophages however it induces only IL-6. Co-stimulatory molecules like CD80 and CD40 were up-regulated in visfatin stimulated monocytes leading to an increased stimulatory capacity in a mixed lymphocyte reaction. Furthermore, visfatin enhanced CD206 mediated phagocytosis in human monocytes and appeared to be a potent chemotactic factor for them (155). Thus one may consider visfatin a potent activator of human monocytes. Whether visfatin modulates macrophage polarization has not been investigated in detail. Recently, it was reported that peritoneal macrophages of visfatin^−/−^ mice showed reduced iNOS expression and NO release after LPS stimulation whereas visfatin treatment of RAW264.7 cells results in an increase of iNOS mRNA and protein expression (160). These finding indicate that visfatin might skew macrophages toward an M1 phenotype.

Very recently, fetuin A has been discovered as a novel adipokine, which could form the link to understand the above discussed conflicting reports concerning the activation of TLR4 by fatty acids. Pal and colleagues assumed that fetuin A is an endogenous ligand through which fatty acids can stimulate TLR4 (161). Fetuin A itself is released from adipocytes in response to excess lipids in an NFκB dependent manner. Furthermore, it is capable to attract macrophages and skew their phenotype from M2 toward M1 polarization as it is described in obesity (161, 162). However, different mechanisms might induce the macrophage phenotype in the creeping fat of CD patients (75).

### Do the answers generated before allow for an explanation of the role of the mesenteric fat tissue within intestinal inflammation? – Consequences for disease

Adipose tissue is a multi potent organ with immune modulating capacity. In CD the normal state of local adipokine, cytokine and cell networks seems to be imbalanced with increased influx of immune cells and altered, usually increased, expression of immune regulating mediators.

**Figure 1:**
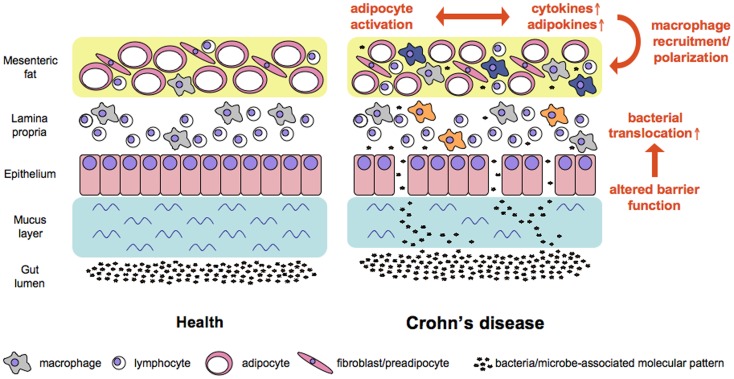
**Working model of factors contributing to increased bacterial translocation and adipose tissue activation in Crohn’s disease**. Alterations in mucus and at the cellular level of the intestinal barrier all contribute to increased translocation of bacterial cells. Bacteria and microbe-associated molecular patterns reach adipose tissue where the subsequent stimulation of pattern recognition receptors affects the ballance of cytokines and adipokines released at this site. In consequence macrophages are recruited and polarized either toward a pro-inflammatory M1 phenotype (e.g., in the lamina propria, orange color) or a regulatory M2 phenotype (e.g., in the mesenteric fat, blue color).

In CD mesenteric fat shows drastic alterations as compared to health with a significant influx of M2-polarized macrophages and decreased adipocytes size. Since most cells present in adipose tissue express innate receptors we assume that the translocation of bacteria and bacterial products that takes place in CD patients is an initial cause for the mesenteric fat alterations. As a consequence of the stimulation via activation of innate receptors the cytokine and adipokine profiles and levels expressed in the mesenteric fat change (see Figure 1). This altered milieu leads to attraction and switch in phenotype of macrophages, an effect that might fuel the local alterations. It might well be that this accumulation of immune cells and their phenotypic conversion represents a protective mechanism of the mesenteric fat to dampen the adjacent inflammation. On the other hand, since pro-inflammatory adipokines are abundant in the tissue another scenario would be, that due to defective response mechanisms following bacterial stimulation the mesenteric fat in CD patients is not a barrier to protect the body from the ongoing intestinal inflammation but involved in the persistence of the intestinal inflammation by increased release of for example leptin and resistin.

Interestingly, some of the factors produced at higher levels in CD fat are abundantly expressed in obesity as well (see Table 1 for alterations in mediator production as compared to health and obesity). Thus, the unique feature of CD mesenteric fat cannot be attributed to abundance of single agents or mediators but seems to be a consequence of the altered proportions of the factors presented in this article.

**Table 1:** **Summarizes alterations in CD adipose tissue as outlined in this review and provides a simplified working model to visualize adipose tissue alterations in CD as compared to normal fat and the state of obesity**.

	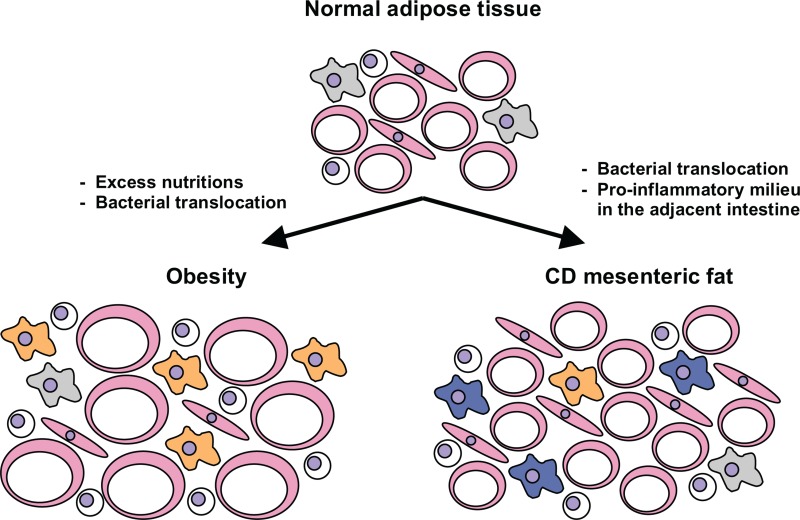		
Cellular alterations	↑	Adipocyte size	↓
	↑	Adipocyte number	↑
	↑	Local immune cells	↑
	↑	M1 macrophages	↑
	–	M2 macrophages	↑
Mediators released	↑	IL-6	–
	↑	TNFα	↑
	↑	MCP-1	–
	n.d.	M-CSF	↑
	↑	Leptin	↑
	↓	Adiponectin	↑
	↑	Resistin	↑
	↑	Visfatin	↑
	↑	C-reactive protein	↑
	—	RANTES	↑
	↑	Free fatty acids	n.d.
	↑	Fetuin A	n.d.
Legend	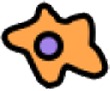 Pro-inflammatory macrophage M1	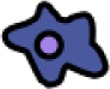 Regulatory macrophage M2
	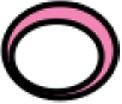 Adipocyte 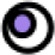 Lymphocyte	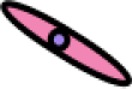 Fibroblast/preadipocyte

*↑↓, increase or decrease as compared to fat from healthy controls; –, no alteration reported; n.d., not done*.

*In general an increase in adipose tissue mass is accompanied by higher production of a broad variety of mediators. Additionally, bacterial products are potent inducers of various adipokines. Several of these mediators attract immune cells, leading to infiltration of the fat by T cells and macrophages. Differences in the relative presence of the various mediators expressed by adipose tissue result in the diverse macrophage phenotypes dominating this compartment. While in obesity pro-inflammatory M1 cells accumulate, in the mesenteric fat of CD anti-inflammatory M2 macrophages increase*.

## Concluding Remarks

With this review we aim to emphasize the critical interaction of the adipose tissue and the immune system. In our view CD as well as obesity form two representative examples. However, we would like to suggest that this interaction is of vital significance for other conditions.

## Author Contributions

Tassilo Kruis, Arvind Batra, and Britta Siegmund developed the structure of the manuscript and contributed equally to the writing part.

## Conflict of Interest Statement

The authors declare that the research was conducted in the absence of any commercial or financial relationships that could be construed as a potential conflict of interest.
